# Material Detection and Sizing of Sub-Resonant Particles by mm-Wave Far-Field Scattering Measurements Using Orthomode Diplexers

**DOI:** 10.3390/s26175406

**Published:** 2026-08-27

**Authors:** Max Lippoldt, Jan Hesselbarth

**Affiliations:** Institute for Radio Frequency Technology (IHF), University of Stuttgart, 70569 Stuttgart, Germany

**Keywords:** electromagnetic scattering, inverse scattering, Mie theory, millimeter wave

## Abstract

**Highlights:**

**What are the main findings?**

- Scattering measurements at millimeter-wave frequencies are employed to classify the material and estimate the size of sub-wavelength-sized particles from several different generic material classes (metal, low/medium/high-permittivity dielectric and liquid).
- Resonant orthomode diplexers are introduced as a novel component for dual-polarized scattering measurements.
- Multi-static measurements are performed to allow for position detection as well as accurate classification and size estimation of displaced particles.

**What are the implications of the main finding?**

- Sub-resonant scattering measurements are shown to be applicable for material detection and size estimation of small particles, e.g., spheres with a radius of 0.4 mm or less at W-band frequencies.
- The presented orthomode diplexers enable dual-polarized scattering measurements from a single electrical port via frequency multiplexing.

**Abstract:**

Bi-static measurements of the scattering of sub-resonant, sub-wavelength-sized spheres and water drops at millimeter-wave frequencies are applied for simultaneous material classification and size estimation. Measurements at Ka-band (26.5–40 GHz) and W-band (75–110 GHz) frequencies are presented that reliably detect the correct material and size of the samples under test. In comparison to traditional resonance-based methods, the sub-resonant approach used here advantageously allows us to characterize smaller particles at a given frequency or to reduce the operating frequency for a given particle size. For the dual-polarized scattering measurements, a novel component (a resonant orthomode diplexer) is proposed. A multi-static measurement is used to additionally determine the position of the sample in the scattering plane. This allows for accurate material classification and size estimation even for a displaced particle. Dielectric and metal spheres with a diameter of 0.4–2 mm are investigated as well as water drops with a volume of 0.5–4 µL.

## 1. Introduction

The simplest three-dimensional shape is arguably a sphere: Its only geometrical parameter is its radius and it has no orientation. Many small particles, such as seeds, dust grains, small liquid drops, etc., can be assumed to have a roughly spherical shape. The scattering of electromagnetic waves by such spherical particles is described by Mie theory [1], the early history of which is described in [2].

Mie theory is widely used for the so-called inverse scattering problem, which involves finding information about the scatterer from the measured scattering. Applications include particle sizing via scattering measurements (spectroscopy) [3,4,5,6], detection of nanoplastics [7], and simultaneous characterization of the size and permittivity of a particle under test for resonant [8] and sub-resonant [9] spheres.

In this paper, building on our previous work [9], the goal is to classify the material of a particle under test (from a known set of materials) and to estimate its size (radius or volume). All measurements are performed in the sub-resonant range. This means that the particles under test are sufficiently small (sub-wavelength size) so that the resonant frequencies of the particles’ eigenmodes [10] are above the considered frequency range. Consequently, the measured scattering of the particles is relatively weak. Therefore, to capture as much information as possible, a dual-polarized measurement is employed. Usually, this would imply the use of orthomode transducers, which are three-port devices, where each of the two single-polarized ports couples to one of the two orthogonal polarizations of the common port (i.e., one port per polarization). Here, instead, novel resonant structures are developed that allow a single port to excite two orthogonal polarizations at different frequencies, so-called orthomode diplexers (OMDs).

A wide variety of generic material classes are examined (low/medium/high-permittivity dielectric, metal, and a liquid drop). Also, the position estimation of a displaced particle under test is explored. Determining the position of a displaced particle from its scattering response allows us to correct the measured scattering accordingly to obtain correct results for the material and size extraction.

The paper is structured as follows: Section 2.1 reviews the scattering of a sphere, discusses the measurement protocol, and introduces the experimental scattering model of a non-spherical water drop. Section 2.2 presents the design of the two OMDs used in this work. Section 2.3 describes the concept used to extract material class and particle size from scattering measurements. Section 2.4 introduces the position estimation and correction of a displaced particle. Section 3.1 and Section 3.2 show the measured results. Lastly, Section 3.3 gives a conclusion and an outlook on future work.

## 2. Materials and Methods

### 2.1. Scattering Model and Measurement Protocol

The electromagnetic scattering of a homogeneous dielectric sphere suspended in free space, illuminated by a plane wave, can be described as [11] (p. 112):

(1) $$\left(\begin{array}{c} E_{||s}\\ E_{⊥s} \end{array}\right)=\frac{e^{jk(r-z)}}{-jkr}\left(\begin{array}{cc} S_{\left|\right|} & 0\\ 0 & S_{⊥} \end{array}\right)\left(\begin{array}{c} E_{||i}\\ E_{⊥i} \end{array}\right)$$

The scattered (*s*) and incident (*i*) electric fields are divided into a parallel and a perpendicular part with respect to the scattering plane, which is defined by the locations of the transmitter, the receiver, and the scatterer. The scattered fields are obtained from the incident fields via the scattering parameters $S_{⊥}$ and $S_{\left|\right|}$ of the sphere under test, which can each be found as an infinite series according to Mie theory [11] (p. 100, 112). For numerical calculations, these infinite series are truncated according to the Wiscombe criterion [12]. Also, note that for a homogeneous isotropic sphere, there is no cross-polarization component of the scattering.

Two parameters govern the scattering of an isotropic sphere in free space: size (radius *a*) and material (complex $\varepsilon_{r}$). In Mie theory, this information is contained in the size parameter x=ka, where *k* is the free-space wave number, and in a contrast parameter (relative refractive index), which for a non-magnetic sphere in free space is expressed as $\sqrt{\varepsilon_{r}}$. A metal sphere is modeled as a purely imaginary permittivity with $\varepsilon_{r}=-j\sigma /\omega \varepsilon_{0}$, where σ is the conductivity.

**Figure 1:**
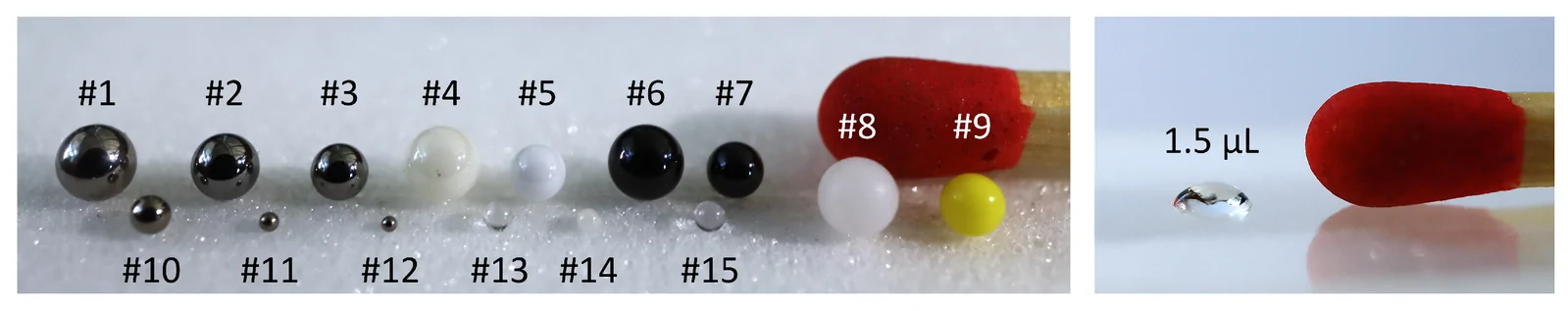
Samples used in this paper: Steel spheres with radii of 1 mm (#1), 0.875 mm (#2), 0.75 mm (#3), 0.5 mm (#10), 0.25 mm (#11), 0.2 mm (#12); alumina spheres with radii of 1 mm (#4), 0.75 mm (#5), 0.4 mm (#13), 0.3 mm (#14); Si_3_N_4_ spheres with radii of 1 mm (#6), 0.75 mm (#7); quartz sphere with radius 0.4 mm (#15); polyoxymethylene (POM) spheres with radii of 1 mm (#8), 0.794 mm (#9). An example of a medium-sized water drop (1.5 µL) is also shown. Match head for size comparison. The suppliers of these spheres are Goodfellow Cambridge Ltd., Huntingdon, UK, Situs Technicals GmbH, Wuppertal, Germany, and HSI-Solutions GmbH, Vienna, Austria.

The samples considered in this paper are shown in Figure 1. Due to their small, sub-wavelength size, the scattering of all samples is measured in the sub-resonant range. For example, the first resonance of the largest alumina sphere (#4) occurs at around 45 GHz and it is consequently sub-resonant at the measurement frequencies in the Ka band (26.5–40 GHz).

**Figure 2:**
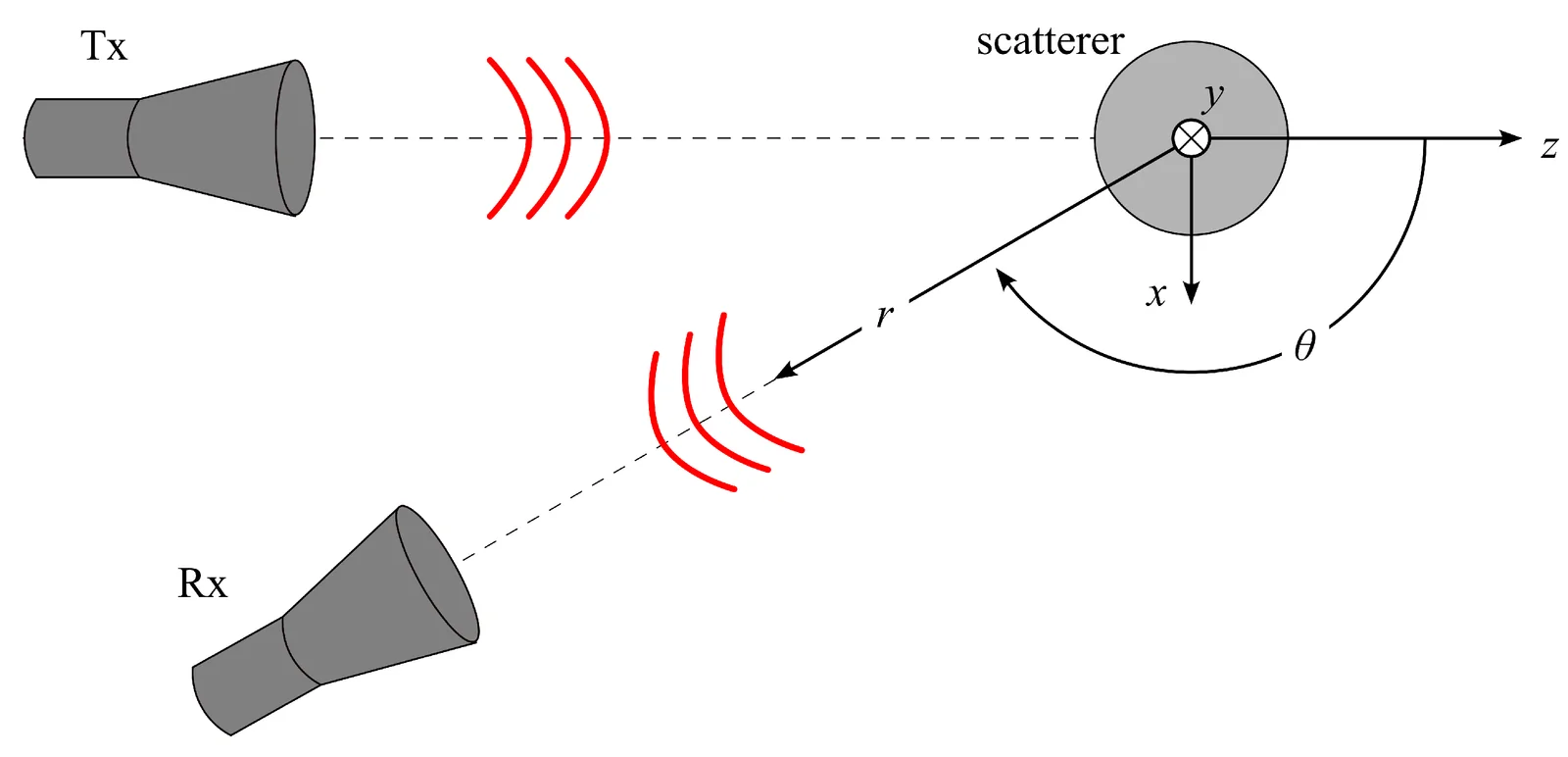
Geometry of the bi-static scattering setup, including the definition of the scattering plane (xz plane), the direction of the incident wave (+z direction) and the scattering angle θ.

A simplified sketch of the measurement setup is shown in Figure 2. To measure $S_{⊥}$, $S_{\left|\right|}$ of a sphere under test, a multi-step approach is employed here, described in more detail in [9]. First, the transmission between the two antennas is measured with a known calibration sphere (#1) using a vector network analyzer (VNA). Then, the sphere is removed from the setup and the transmission without any sample is recorded. Lastly, the sample under test is placed in the setup and the transmission is recorded again. Each measurement is performed for both polarizations, using OMDs (i.e., $S_{⊥}$ and $S_{\left|\right|}$ are obtained for different frequency ranges). By subtracting the transmission without a sample from the transmission with the sample under test and then multiplying the result with a calibration factor obtained from the measurement of the known calibration sphere, the scattering parameters $S_{⊥}$, $S_{\left|\right|}$ of the sphere under test are obtained. Note that Mie theory as described in [11] and in (1) uses the convention $e^{-j\omega t}$ instead of $e^{+j\omega t}$. A plane wave propagating in the +z direction is accordingly written as $e^{-j\omega t}e^{+jkz}e^{j\varphi }$. For that reason, complex conjugation of the (complex, linear) transmission parameters measured by the VNA is necessary to match the theoretical calculations. All calculations and data analyses are performed using MATLAB (version R2024b). Field simulations are carried out in CST Microwave Studio.

Besides the mechanically well-defined dielectric and metal spheres, water drops (tap water) are also considered in this work. The drop volumes of 0.5–4 µL are chosen to be in the same range as the volume of spheres with radius 0.5–1 mm. The volume of the drops is controlled by a generic air displacement pipette. By placing the drop on a thin plastic strip with an indentation, a spherical shape is (rather crudely) approximated. Measurements reveal, however, that the measured scattering especially of the larger drops deviates drastically from the one predicted by Mie theory, assuming a permittivity of water according to [13]. This can be attributed to the fact that the shape of the larger drops deviates more from an ideal sphere. Consequently, in this work, a different scattering model is introduced for the water drops, as the Mie theory model with its spherical shape assumption is too inaccurate.

**Figure 3:**
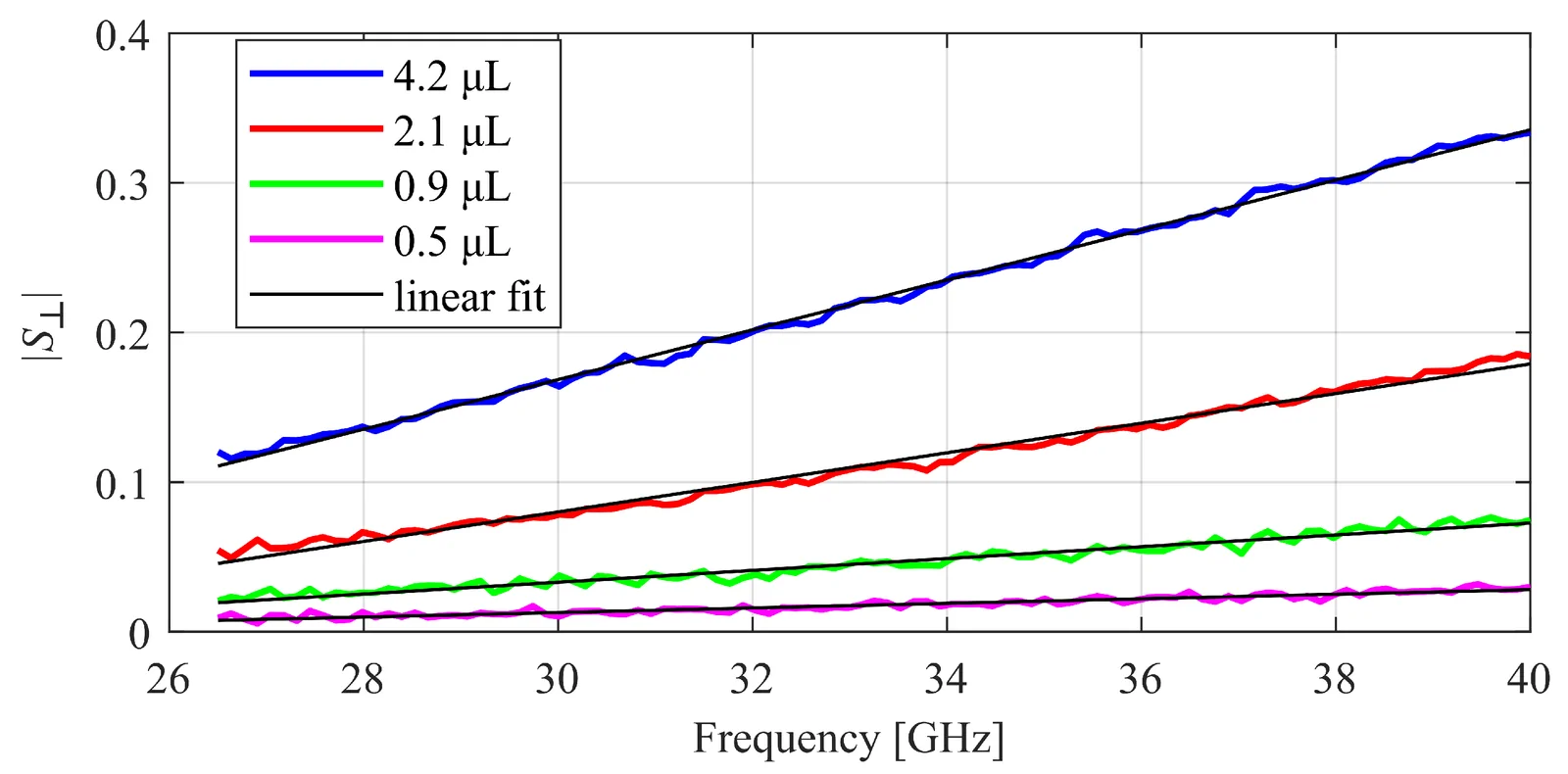
Scattering of water drops, exemplarily shown for $|S_{⊥}|$: Measured scattering of four different drop volumes versus linear fit.

As an alternative, it is found that the scattering curves over frequency of the water drops are very well approximated by a linear fit, as exemplarily shown for $|S_{⊥}|$ in Figure 3. This linear fit is of the form $S=cf+S_{0}$, where *S* is the scattering parameter ($S_{⊥}$ or $S_{\left|\right|}$) and *f* is the frequency. For each polarization, the two complex-valued parameters *c*, $S_{0}$ are functions of the drop volume *V*. These functions c(V) and $S_{0}\left(V\right)$ are sampled with the four measurements of Figure 3 and each approximated as a second-order polynomial. This generates an experiment-based model of the scattering of a water drop that relates the drop volume to the two parameters of a linear curve and via that to the scattering parameters $S_{⊥}$ and $S_{\left|\right|}$. The applicability of this simple model is proven by the extracted results shown in Section 3.1.

### 2.2. Orthomode Diplexers

To measure the dual-polarized scattering with only one electrical port per scattering angle, special resonant structures are developed that we term OMDs. They couple a single-polarized input port (rectangular waveguide) to either one of the two orthogonal polarizations of a dual-polarized output port (dual-linearly polarized antenna) depending on the operating frequency so that the vertical output polarization is excited for a certain frequency range whereas the horizontal polarization is excited for a different frequency range. By that frequency multiplexing, these structures allow for faster dual-polarized scattering measurements and avoid the need to mechanically alter the measurement setup as in previous work [9], thereby reducing misalignment errors. Also, for a VNA with a given number of ports, OMDs allow for the simultaneous measurement of more scattering angles: e.g., a four-port VNA could connect to two orthomode transducers (=2 dual-pol. antennas) versus four OMDs (=4 dual-pol. antennas). Electrical switching could also be an option, but would increase the measurement time and degrade the signal-to-noise ratio. Principally, the desired behavior of an OMD could be achieved using a classical orthomode transducer (e.g., [14,15,16]) together with a diplexer filter (e.g., [17,18,19]). However, the resonant OMDs presented here are much more compact compared to that generic structure. Note also that somewhat similar structures with significantly higher electrical and mechanical complexity exist for satellite communication applications [20,21,22,23,24].

Generally, the information about the material and size of the particle under test is contained within the value of the (complex) scattering parameters $S_{⊥}$, $S_{\left|\right|}$ as well as their behavior over frequency. To obtain as much information as possible, therefore, the scattering should be measured for both polarizations and over a wide frequency bandwidth (cp. [9]). With resonant OMDs, a wide-band measurement of both $S_{⊥}$, $S_{\left|\right|}$ is principally difficult. However, for sub-resonant particles, no resonant peaks exist in the scattering spectrum and the scattering curves are monotonous over frequency (almost linear for small particles). In this case, most of the information about the behavior over frequency of the scattering of a certain particle can be obtained from the scattering parameter values at the lowest and highest considered frequency. This means that these two frequency points already contain most of the information about the wide-band scattering spectrum of the sub-resonant particle. Thus, instead of a wide-band measurement, a large frequency spacing for the resonances corresponding to the two orthogonal polarizations is chosen. This way, the measured information about the frequency behavior of the scattering is maximized even without a wide-band measurement. Note that here, due to the rectangular waveguide input port of the OMDs, the maximum frequency spacing is limited to approximately half an octave.

**Figure 4:**
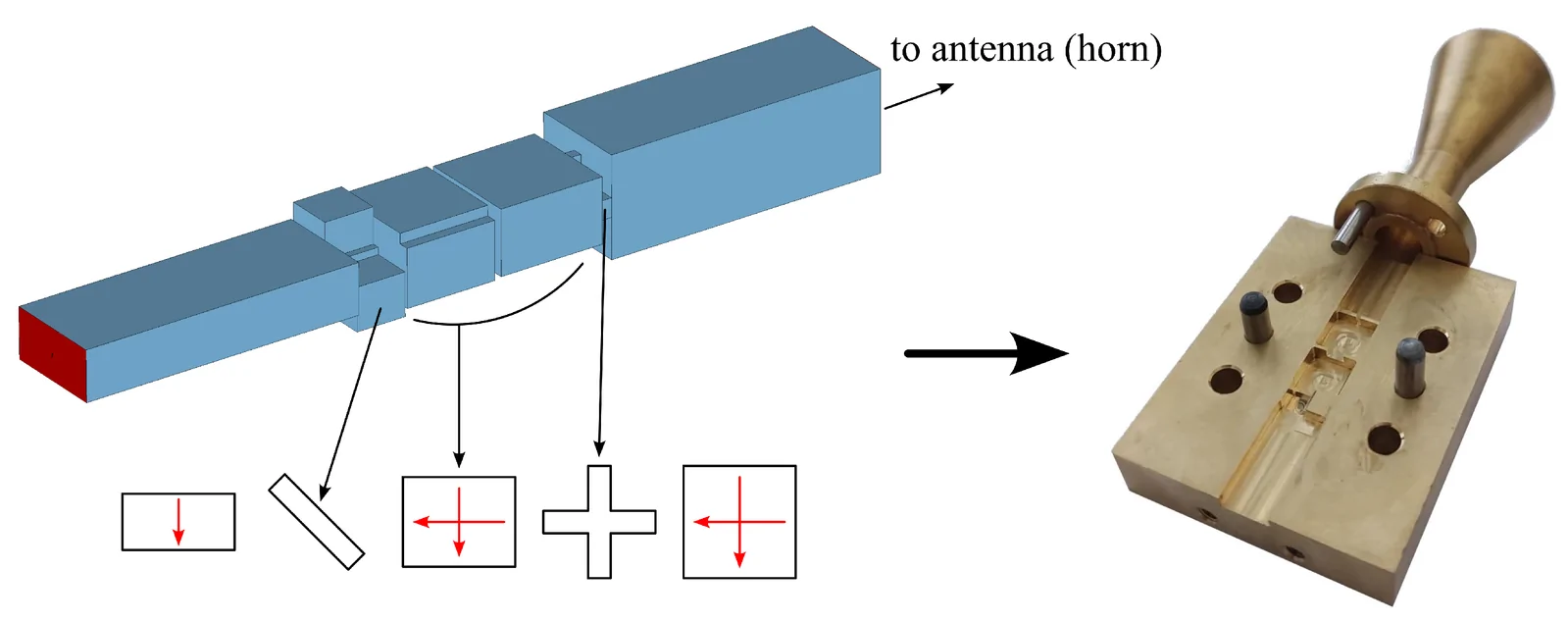
Structure, working principle, and fabricated prototype of a dual-resonant waveguide OMD working in the Ka band. Top half of fabricated structure removed for clarity.

The first OMD is designed for Ka band frequencies as a rectangular waveguide structure. Its geometry is shown in Figure 4. The separation of the polarization over frequency is achieved by two dual-resonant cavities. A standard WR-28 waveguide feeds the structure. A diagonal iris (realized in stepped form for ease of fabrication) generates a tilted electric field that couples to both the vertical and horizontal resonance mode of the adjacent cavities. By using two slightly differently dimensioned cavities, two separate resonances are achieved per polarization and thus a relatively wide frequency bandwidth for each polarization. The first cavity uses steps on two of its four longitudinal edges to counter the tilt of the electric field in the resonator caused by the diagonal iris, thereby reducing the cross-polarization coupling. A cross-shaped iris is used to couple the resonant cavities to the dual-polarized square waveguide.

The OMD is fabricated as a split-block design. At the square waveguide port, a circular horn antenna is placed. The circular shape allows for relatively easy fabrication by precision turning as opposed to a square pyramidal horn antenna. To test the system performance (OMD + antenna), S parameter measurements are performed with a Keysight N5225B VNA with the antenna connected to the dual-polarized port of the OMD and radiating into free space. For transmission measurements, to determine the cross-polarization level of the system, a second antenna (single-polarized standard gain horn) is placed in the far field and manually rotated by 90°. For the vertical output polarization, the OMD–antenna system exhibits a measured input reflection $S_{11}$ of better than −6 dB for 29.4–30.4 GHz and a measured radiated cross-polarization of better than −25 dB in the forward direction. For the horizontal output polarization, the measured $S_{11}$ is better than −10 dB for 38.0–39.0 GHz with a measured cross-polarization of better than −22 dB. At 29.9 GHz, the simulated antenna gain is 16.9 dBi with a measured half-power beam width (HPBW) of 25.2° (E plane, simulated 24.4°) and 31.2° (H plane, simulated 29.6°). At 38.5 GHz, the simulated gain is 18.8 dBi and the following HPBWs are measured: 19.5° (E plane, simulated 19.4°) and 24.0° (H plane, simulated 23.6°). Overall, the measured performance agrees well with the simulated values, although the measured input reflection for the lower resonance deviates from the simulation. This can be attributed to the imperfect contact of the two halves of the split block.

**Figure 5:**
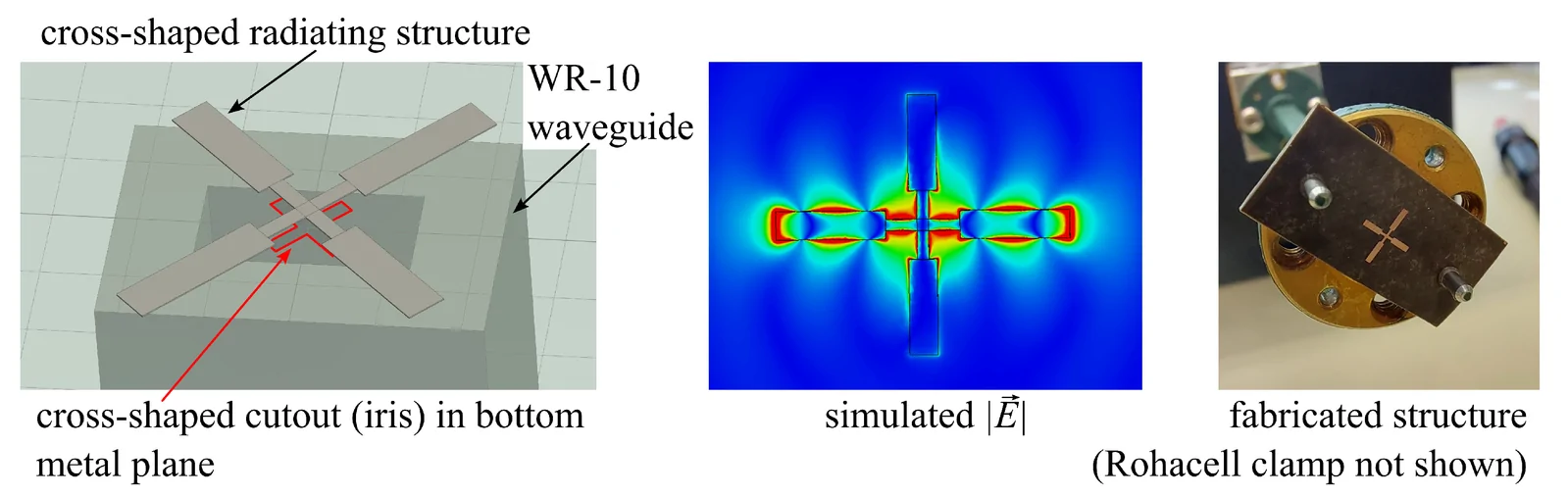
Structure, simulated electric field, and fabricated prototype of a cross-shaped patch OMD working in the W band.

As a proof of concept at higher frequencies, a second OMD is designed for operation in the W band. Its structure is shown in Figure 5. In this case, the polarization separation is achieved by a dual-resonant antenna that can be understood as a cross-shaped patch antenna (or as a pair of crossed dipoles). The arms of the cross have different lengths and thus different resonant frequencies. Because the radiated wave is polarized along the arm that is in resonance, this structure achieves frequency-dependent dual-polarized operation. The cross structure is printed on the top metal layer of a thin circuit board and fed by a WR-10 waveguide through a cross-shaped slot in the bottom metal layer of the board. Overall, the functional principle is roughly the same as for the Ka-band OMD: a 45° tilted iris (in the board’s ground plane) couples to two geometrically orthogonal resonance modes, which are controlled by the length of the “arms” of the cross-shaped patch. The resonant field pattern of one arm is shown in Figure 5. To obtain good radiation and matching of the structure while also sufficiently separating the two polarizations (i.e., the resonant fields of the two arms), the patch was dimensioned larger than usual so that each arm does not resonate in a half-wavelength mode but in a 1.5-wavelength manner. The antenna is fabricated on 0.254 mm Rogers 5880. To clamp the circuit board to the WR-10 flange and enforce electrical contact between the board ground plane and the flange, a holder made from Rohacell foam is used for this prototype.

For the vertical output polarization, the measured $S_{11}$ of the fabricated antenna is better than −6 dB for 93.8–95.8 GHz with a measured (radiated) cross-polarization of better than −10.7 dB in the forward direction. For the horizontal polarization, the measured $S_{11}$ is better than −6 dB for 103.5–105.5 GHz with a measured cross-polarization of better than −13.5 dB. The simulated antenna gains are 8.6 dBi (V polarization) and 10.6 dBi (H polarization). Note that in this case, the less-than-ideal performance of the dual-resonant antenna OMD is accepted as a tradeoff for simple fabrication given the proof-of-concept nature of the W-band measurements.

### 2.3. Classification and Sizing

**Figure 6:**
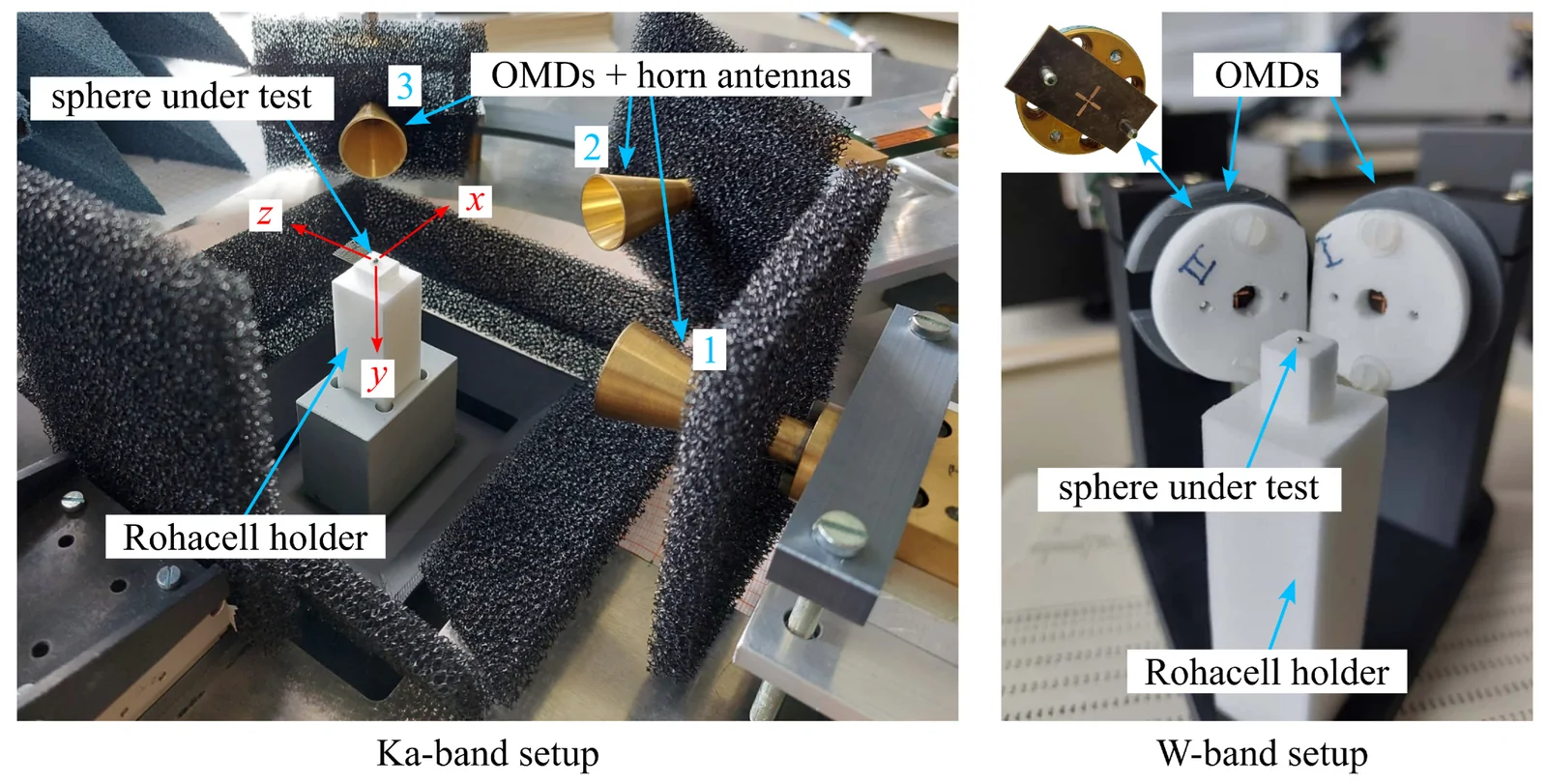
Photo of the measurement setups for the Ka and W band.

The fabricated OMDs in their corresponding scattering measurement setup are shown in Figure 6. Note that for the Ka-band setup, in this section, only antennas 1 and 2 are used (antenna 3 is used for the position correction as described in Section 2.4). As outlined in [9], a scattering angle of 120° is chosen. The antenna–sample distance is set to 80 mm (Ka band) and 25 mm (W band) to ensure far-field conditions. As samples, several generic material classes are used here (cp. Figure 1): low-, medium-, high-permittivity low-loss dielectrics, conductive metal (steel), and a liquid drop (tap water). The following values are assumed for the material classification: relative permittivities of 2.9 for POM, 3.8 for quartz, 8.7 for Si_3_N_4_, and 10 for alumina, all with tan δ=0; conductivity of 1.35 MS for steel. Small changes in permittivity (i.e., a sample permittivity deviating slightly from the assumed permittivity of the material class) lead to an almost negligible change in estimated radius. With similar reasoning, the permittivities are assumed to be constant over the considered frequency ranges. Additionally, the dielectric spheres are assumed to be homogeneous and isotropic. Small inhomogeneities in the permittivity could be considered via an effective permittivity, averaged over the volume of the corresponding sphere. Note that the water drop scattering is calculated via an experiment-based model (cp. Section 2.1) that implicitly contains the water’s permittivity variation over frequency. All measurements are performed with a Keysight N5225B VNA.

**Figure 7:**
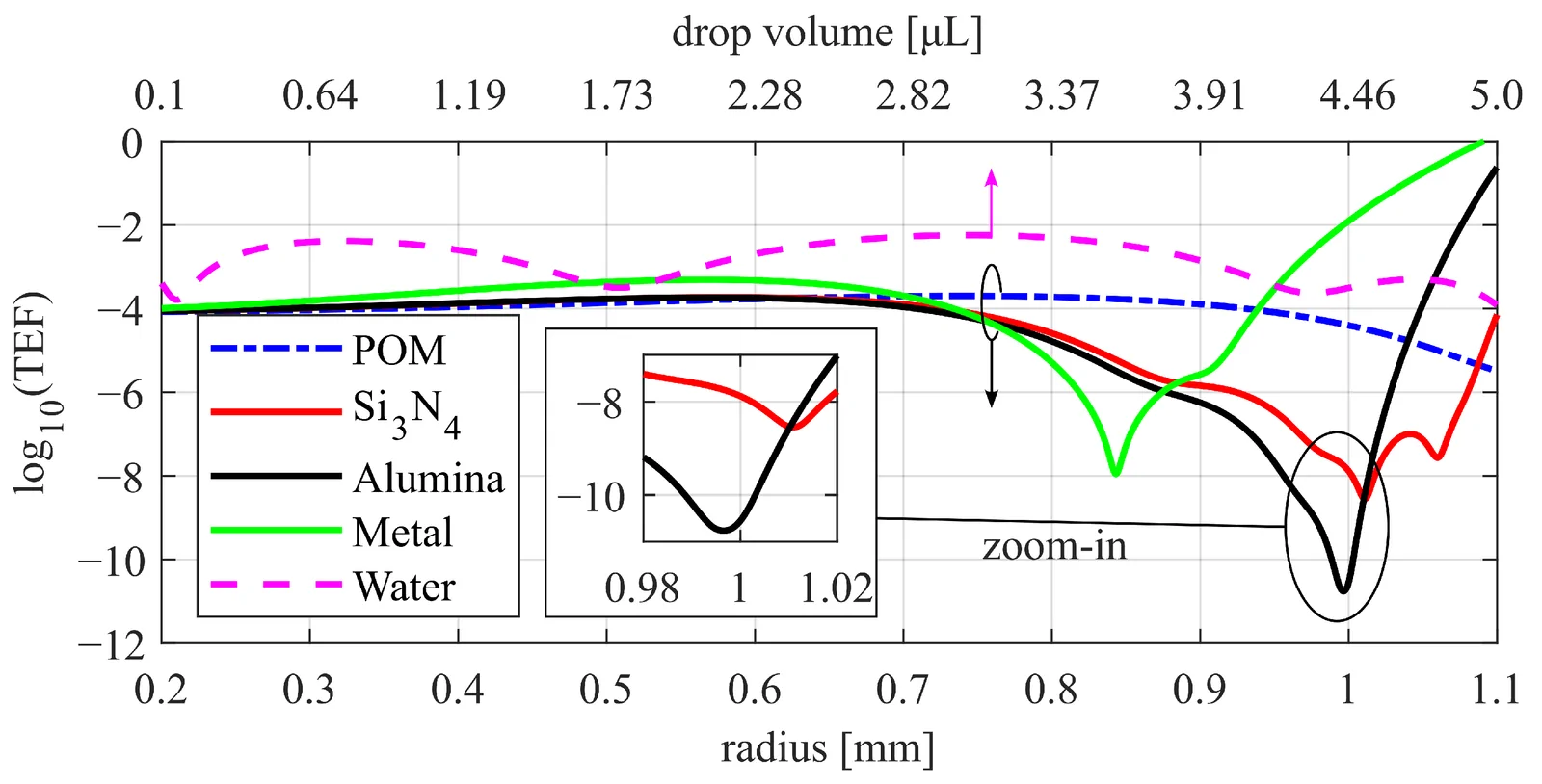
Plot of the total error function (TEF) for sample #4. The material and radius combination that minimizes the TEF is taken as the extracted value for that specific sample (here: Alumina—0.996 mm, cp. Table 1).

After obtaining the measured $S_{⊥}$, $S_{\left|\right|}$ as outlined in Section 2.1, the material and size are found by comparison of the measured curves (real and imaginary parts of $S_{⊥}$ and $S_{\left|\right|}$ over frequency) with a set of library functions calculated using Mie theory and the experiment-based water drop scattering model outlined in Section 2.1, respectively. These library functions are calculated for a large number of combinations between material class and size (five material classes for the Ka band, three material classes for the W band, and 1501 size or volume values for each material class). The best-fitting library function (in a least-squares sense) then yields the extracted material and size of the sample under test. This approach is discussed in detail in [9] and has conceptually been used in the literature before (e.g., in [25]). As a measure of how well a certain library function matches with the measured scattering curves, a total error function (TEF) value is calculated via error square sums over frequency (for exact formulations, see [9]). The best-fitting library function is consequently found by the minimum of the TEF over all considered library functions (i.e., all considered material and size combinations) as exemplarily shown for sample #4 in Figure 7. As shown, there is a clear distinction between the minima of the different material classes. Here, the (correct) alumina class achieves the lowest minimum TEF magnitude, whereas the minimum of the second-best-fitting Si_3_N_4_ class is two orders of magnitude larger and the minima of the other material classes are even further separated. The corresponding results for all samples are presented in Section 3.1.

### 2.4. Position Correction

In the previous sections, it was assumed that the particle under test is exactly in the center of the xz plane (cp. Figure 2). In the measurement procedure, this position is defined by the location of the calibration sphere during its measurement. A microscope is used to optically confirm the correct positioning of the sample. If a sample is displaced in the xz plane, however, the resulting phase offset of the measured scattering can lead to incorrect results (e.g., #4 placed at x= 0 mm, z= 1 mm is recognized as alumina with 0.785 mm radius).

**Figure 8:**
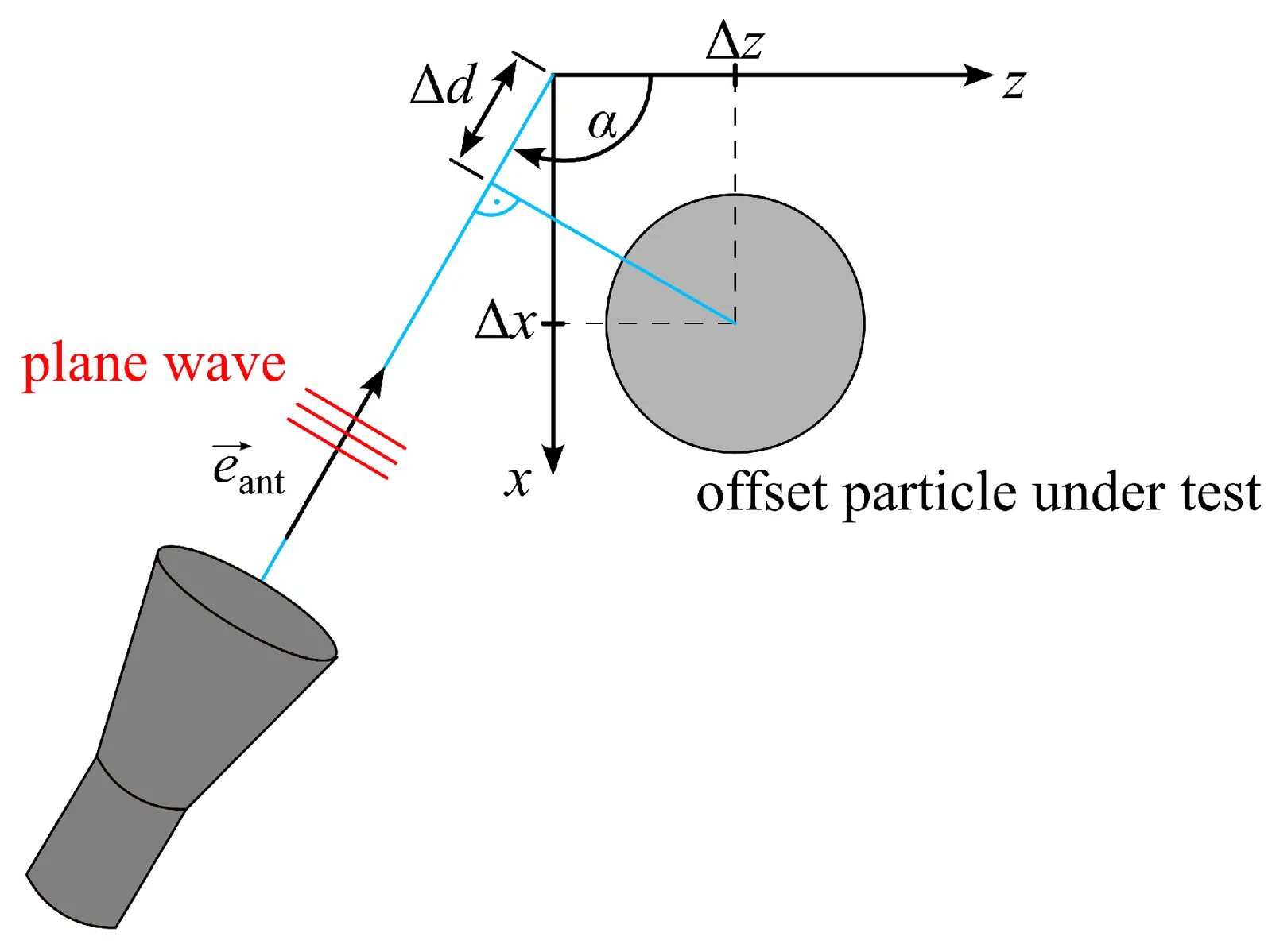
A displaced particle leads to a change in the path length that the plane wave has to travel from the antenna to the particle (or vice versa). The origin of the xz plane is defined by the location of the calibration sphere during its measurement.

To analyze the effect of a displaced particle on the phase of the measured scattering, plane waves are assumed. Simulations show that for the Ka band setup, 80 mm from the antenna aperture, the phase of the radiated wave varies less than ±0.75° from constant (ideal plane wave) over a range of ±2 mm in the transverse direction around the center of the wave. This deviation is considered negligible. Under this approximation, an offset in the *y* direction does not significantly change the measured scattering, which was also confirmed experimentally [9]. A displacement in the xz plane, however, leads to a change in path length as shown in Figure 8. The projection of the displacement (Δx,Δz) onto the propagation direction ${\vec{e}}_{\mathrm{ant}}$ given by the antenna orientation (angle α) yields a change in path length Δd as follows:

(2) Δd=−Δxsinα−Δzcosα

Note that Δd is defined such that a negative value indicates a shorter path length. Considering the time dependence of $e^{-j\omega t}$, as mentioned above, the corresponding one-way (i.e., antenna-to-particle or particle-to-antenna) phase shift generated by this change in path length is Δφ=2πΔd/λ, where λ is the free-space wavelength. The unambiguous range of Δφ limits the range of the detectable position offset to $|\Delta d_{\max}|\;=\lambda /2$ at the highest considered frequency while the practically examined offsets in this work are much smaller. Note also that the angle α (related to the measurement setup) is used here instead of the scattering angle θ (cp. Figure 2) because θ, according to Mie theory, is defined from the propagation direction of the plane wave radiated by the transmit antenna.

To make the measurement and extraction procedure more robust against particle displacements, it is necessary to first find the exact position of the sample and then to correct the phase of its measured $S_{⊥}$, $S_{\left|\right|}$ accordingly before performing the classification and sizing step. This is possible by incorporating a third OMD-fed antenna into the setup (antenna 3 in Figure 6), which in this case is placed at α= 50° in addition to the two other antennas at α= 120° and 180°. Because of the rotational symmetry of a sphere, the backward scattering to each of the three antennas by the sphere under test has the same phase if the sphere is centered correctly (and after performing the calibration protocol described above). From the two phase differences between the three measured backward scatterings, the xz position of the sphere under test can thus be found without prior knowledge of the sphere’s material or size. The required calculations for this triangulation step are trivial and are omitted here. The corresponding results are presented in Section 3.2.

## 3. Results and Discussion

### 3.1. Classification and Sizing Results

The extracted results using the Ka-band OMDs (cp. Figure 6, using only antennas 1 and 2) are shown in Table 1. The detected material classes are correct in each case and the extracted radii and volumes agree well with the mechanically measured radii and dispensed volumes. To further investigate the reliability of the measured results, 11 consecutive measurements are performed for both the largest metal sphere (#1) and the smallest low-permittivity POM sphere (#9). Between each measurement, the sphere is removed from the holder and then placed again. In all cases the material class is detected correctly. For the metal sphere, the mean of the extracted radii is 1.0014 mm, varying between 0.9992 and 1.0034 mm with an empirical standard deviation of 1.4 µm. For the POM sphere, the mean of the extracted radii is 0.7851 mm, varying between 0.7820 and 0.7886 mm with an empirical standard deviation of 2.3 µm (cp. Table 1). These results confirm the repeatability and reliability of the proposed measurement.

The extracted results using the W-band OMDs (cp. Figure 6) are shown in Table 2. A good agreement between the mechanically measured and extracted values is also observed here. This demonstrates that the measurement principle can easily be frequency-scaled to classify and size relatively smaller particles.

### 3.2. Position Correction Results

To validate the position correction concept, test measurements with spheres #1 and #4 at different xz positions are performed, using the Ka-band setup as shown in Figure 6 (using all three antennas). In all cases, with position estimation and correction, the material and size of the sphere under test are detected correctly. The extracted positions are shown in Table 3. The given mean values and confidence intervals are calculated over all frequency samples, considering that each single frequency sample returns a measured value for the sphere position. The results are in good agreement with the approximate positions of where the spheres were placed, considering that the placement was performed using manual alignment (estimated accuracy ±0.1 mm) with the help of a microscope. Also, the calculated confidence intervals compare favorably with the estimated accuracy of the manual alignment.

### 3.3. Discussion

In this work, the simultaneous material classification and size estimation of various samples was successfully demonstrated for sub-wavelength-sized, sub-resonant particles by using far-field scattering measurements. Additionally, a method for estimating the position of a displaced sphere under test and correcting the measurements accordingly was introduced. For the dual-polarized scattering measurements, newly developed structures (OMDs) were presented and used. The extracted values agree well with the expected and mechanically measured values.

Future work could use the concept presented here to build a scattering measurement setup that allows us to classify and size samples through electromagnetically transparent packaging. Also, the detection and volume estimation of falling drops of liquid could be explored. Furthermore, to enhance the signal-to-noise ratio by capturing more of the weak scattering of the sub-resonant particle, the antenna–sample distance could be reduced. This would have two implications: First, the theoretical scattering model would need to be modified to account for spherical wave fronts. Second, for a given antenna–sample distance, a horn antenna might not be the best choice with respect to the generated field strength at the particle location and the development of a specialized antenna could offer potential for further improvement [26].

## Figures and Tables

**Table 1:** Extracted results: Ka band.

	Mechanically Measured	Extracted
Sample	Radius or Dispensed Volume	Material and Size
#1	0.998 mm	Metal—1.001 mm (σ= 0.0014 mm)
#2	0.876 mm	Metal—0.878 mm
#3	0.750 mm	Metal—0.754 mm
#4	0.999 mm	Alumina—0.996 mm
#5	0.749 mm	Alumina—0.751 mm
#6	0.999 mm	Si_3_N_4_—0.998 mm
#7	0.749 mm	Si_3_N_4_—0.752 mm
#8	0.985 mm	POM—0.985 mm
#9	0.792 mm	POM—0.785 mm (σ= 0.0023 mm)
Water drop	4 µL	Water—4.059 µL
Water drop	3 µL	Water—2.811 µL
Water drop	2 µL	Water—1.991 µL
Water drop	1.5 µL	Water—1.361 µL
Water drop	1 µL	Water—0.884 µL
Water drop	0.5 µL	Water—0.672 µL

**Table 2:** Extracted results: W band.

	Mechanically Measured	Extracted
Sample	Radius [mm]	Material and Size
#10	0.499	Metal—0.503 mm
#11	0.249	Metal—0.265 mm
#12	0.201	Metal—0.207 mm
#13	0.396	Alumina—0.406 mm
#14	0.300	Alumina—0.289 mm
#15	0.399	Quartz—0.414 mm

**Table 3:** Extracted position: Ka band.

	Placed at	Extracted	Extracted
Sphere	Δx, Δz[mm]	Δx [mm] *	Δz [mm] *
#1	0, 0	−0.005 ± 0.034	−0.027 ± 0.010
#1	0.5, 0.5	0.561 ± 0.056	0.519 ± 0.021
#1	0, 1	−0.003 ± 0.044	1.004 ± 0.012
#1	1, 0	1.056 ± 0.045	0.029 ± 0.009
#4	0, 0	0.040 ± 0.059	−0.019 ± 0.029
#4	0.5, 0.5	0.600 ± 0.072	0.494 ± 0.032
#4	0, 1	0.021 ± 0.077	0.992 ± 0.033
#4	1, 0	1.064 ± 0.075	0.054 ± 0.048

* including 95% confidence interval.

## Data Availability

The original contributions presented in this study are included in the article. Further inquiries can be directed to the corresponding author.
